# MicroRNA-363 negatively regulates the left ventricular determining transcription factor *HAND1* in human embryonic stem cell-derived cardiomyocytes

**DOI:** 10.1186/scrt464

**Published:** 2014-06-06

**Authors:** Vilas Wagh, Alexander Pomorski, Karlijn J Wilschut, Sebastian Piombo, Harold S Bernstein

**Affiliations:** 1 Eli & Edythe Broad Center of Regeneration Medicine and Stem Cell Research, Department of Pediatrics, University of California San Francisco, 500 Parnassus Avenue, San Francisco, CA 94143, USA; 2 Current address: Merck Sharp & Dohme Corp., Whitehouse Station, NJ, USA

## Abstract

**Introduction:**

Posttranscriptional control of mRNA by microRNA (miRNA) has been implicated in the regulation of diverse biologic processes from directed differentiation of stem cells through organism development. We describe a unique pathway by which miRNA regulates the specialized differentiation of cardiomyocyte (CM) subtypes.

**Methods:**

We differentiated human embryonic stem cells (hESCs) to cardiac progenitor cells and functional CMs, and characterized the regulated expression of specific miRNAs that target transcriptional regulators of left/right ventricular-subtype specification.

**Results:**

From >900 known human miRNAs in hESC-derived cardiac progenitor cells and functional CMs, a subset of differentially expressed cardiac miRNAs was identified, and *in silico* analysis predicted highly conserved binding sites in the 3′-untranslated regions (3′UTRs) of Hand-and-neural-crest-derivative-expressed (*HAND*) genes 1 and 2 that are involved in left and right ventricular development. We studied the temporal and spatial expression patterns of four miRNAs in differentiating hESCs, and found that expression of miRNA (miR)-363, miR-367, miR-181a, and miR-181c was specific for stage and site. Further analysis showed that miR-363 overexpression resulted in downregulation of *HAND1* mRNA and protein levels. A dual luciferase reporter assay demonstrated functional interaction of miR-363 with the full-length 3′UTR of *HAND1*. Expression of anti-miR-363 *in-vitro* resulted in enrichment for *HAND1*-expressing CM subtype populations. We also showed that BMP4 treatment induced the expression of *HAND2* with less effect on *HAND1*, whereas miR-363 overexpression selectively inhibited *HAND1*.

**Conclusions:**

These data show that miR-363 negatively regulates the expression of *HAND1* and suggest that suppression of miR-363 could provide a novel strategy for generating functional left-ventricular CMs.

## Introduction

Heart cells are unable to repair after damage, which ultimately leads to more than 5 million deaths per year worldwide due to heart failure [1]. The past few decades have witnessed new therapeutic options for treating diseases that are caused by cell damage; however, the prevalence of heart failure continues to increase [2]. Repair and regeneration of lost cardiac cells either with endogenous cells (direct reprogramming) or by using cell-based therapies (cell transplant) holds great promise, but obstacles must be addressed before widespread clinical use is adopted. For example, the cardiac cells obtained after differentiation of pluripotent stem cells comprise mixed cardiomyocyte (CM) populations with heterogeneous mechanical and electrical properties that may be more or less useful for transplantation [3].

The Hand-And-Neural-crest-Derivative-expressed (HAND) superfamily of class B basic helix-loop-helix factors consists of two members, *HAND1* and *HAND2*, both of which are dynamically expressed in embryologically distinct lineages during development, and have been shown to play essential roles in the developing heart. To date, the precise mode of function for either HAND protein remains unknown, although many studies collectively suggest that these factors play roles in activating multiple genes and that the mechanism of their regulation is complex [4,5]. Although the progenitor cells and general timing of myocardial differentiation have been determined, little is known about the mechanisms controlling commitment of progenitors and the maturation of myogenic cells that give rise to atrial, ventricular, and specialized-conduction CMs. Although it is clear that heterogeneous CM populations arise during stem cell differentiation [6], mechanisms that control the cellular subspecification of cardiogenic mesoderm remain obscure.

Small, noncoding microRNAs (miRNAs) are emerging as important posttranscriptional regulators of gene expression, with each miRNA predicted to regulate hundreds of mRNA target genes [7,8]. miRNAs have tissue-specific distributions that play key roles in cellular physiology, such as cell proliferation, differentiation, and death [9]. miRNAs also are known to influence biologic and metabolic processes that are dysregulated in various diseases [10]. To explore their role in normal development and disease, we and others have described miRNA expression profiles that are informative about cardiogenesis and skeletal muscle differentiation [11-13].

As miRNAs function mainly through the inhibition of target genes, we analyzed the expression of these oligomers in hESC-derived cardiac cell populations with the intention of identifying miRNAs involved in CM-subtype specification, in particular left ventricular CMs. We identified four miRNAs (miR-363, -367, -181a, and -181c) that putatively target *HAND1* and/or *HAND2*. Of these miRNAs, we showed that miR-**363** specifically targets *HAND1* during CM-subtype specification in hESCs.

## Methods

### Cell culture and differentiation

The UCSF Stem Cell Research Oversight Committee approved all experiments with hESCs. H9 hESC (WA09, WiCell) and BCiPSP16 human induced pluripotent stem cells (hiPSCs, a gift from B. Conklin, Gladstone Institutes, UCSF) were passaged as undifferentiated cells on irradiated mouse embryonic feeder cells (MEFs) in Hes medium (Knockout-DMEM-F12, 20% Knockout-Serum-Replacement, 1% nonessential amino acids, 0.1 m*M* β-mercaptoethanol (all purchased from Invitrogen, Carlsbad, CA, USA) supplemented with 15 ng/ml Basic fibroblast growth factor-2 (FGF-2; R & D Systems, Minneapolis, MN, USA)) as previously described [14]. Alternatively, cells were cultured in feeder-independent conditions on plates coated with Matrigel (BD Biosciences, Bedford, MA, USA), in mTeSR medium (Stem Cell Technologies, Vancouver BC, Canada), according to manufacturer’s instructions.

Differentiation was initiated by passaging hESCs or hiPSCs onto low-attachment plates (Corning Inc., Acton, MA, USA) in differentiation medium consisting of Knockout-DMEM-F12, 20% Fetal Bovine Serum, 1% nonessential amino acids, and 0.1 m*M* β-mercaptoethanol. Medium was replenished every second day.

### Isolation of differentiated cell populations

hESC- and hIPSC-derived cells were isolated on alternate days (2, 4, 6, 8, 10, 12, and 14 days) after differentiation. Additionally, all beating foci from 12 and 14 days were microdissected and collected as representative samples of CM subtypes.

### miRNA inhibition and overexpression

Pre- and anti-miRNAs were purchased from Ambion (Austin, TX, USA). Transfections were performed with Lipofectamine (RNAimax; Invitrogen, Carlsbad, CA, USA) according to the manufacturer’s instructions. Transfection complexes were prepared with 50 to 100 n*M* pre- or anti-miRNA, and cells were transfected 24 hours after plating.

### Fluorescent *in situ* hybridization

Fluorescent *in situ* hybridization (F-ISH) with double-Dig-labeled miR-363 miRCURY LNA probes (Exiqon, Copenhagen, Denmark) on *E*10.5 mouse embryos was performed as described in the manufacturer’s protocol. In brief, slides were treated with PCR-grade proteinase-K (Roche Diagnostics, Mannheim, Germany) after fixation. The hybridization mix was prepared with 20 pmol of miR-363 double-labeled LNA probes in hybridization solution. The hybridization temperature used was 15°C below the melting temperature of the miR-363 probe.

### Quantitative real-time PCR

Total RNA was extracted by using the mirVana RNA isolation kit (Ambion, Austin, TX, USA). miRNA expression was analyzed by mirVana reverse quantitative transcription-PCR miRNA detection assay according to the manufacturer’s protocol. In brief, cDNA was converted from 10 ng of total RNA by using miRNA-specific primers with the multiScribe reverse transcription kit (Ambion, Austin, TX, USA). cDNA was diluted 15 times with nuclease-free water, and 5 μl was used as a template for PCR. Quantitative real-time reverse transcriptase PCR (qPCR) was carried out on an Applied Biosystems 7300 cycler. Relative miRNA levels were calculated by using the ∆∆Ct method and represented relative to housekeeping miRNA (RNU48) expression. For mRNA expression analysis, the Taqman assay was carried out per the manufacturer’s instructions. The data were represented as relative quantitation with *GAPDH* as an internal control.

### 3′UTR reporter assay

The full-length 3′UTRs of human *HAND1*, *HAND2,* and *NKX2.5* were each inserted downstream of the Firefly luciferase gene in *pEZX-MT01* (GeneCopoeia, Rockville, MD, USA). *Renilla* luciferase encoded by the same vector was used as transfection control in the Dual-Luciferase Assay (Promega, Madison, WI, USA). hESCs were plated in a 96-well plate and co-transfected with 50 ng of luciferase vector and 50 n*M* precursor by using lipofectamine. Dual-Luciferase assays were performed according to the manufacturer’s instructions on a Wallac-Trilux luminometer. Luciferase assays also were performed in HEK293 cells. The luciferase assay kit (Promega Inc., Madison, WI, USA) was used to measure the reporter activity according to the manufacturer’s instructions.

### Immunoblot analysis

Immunoblot analysis was performed as previously described [14]. Cell lysates were separated with SDS-PAGE in 10% polyacrylamide gels and transferred onto nitrocellulose membranes. After blocking of nonspecific binding sites for 2 hours with 5% nonfat milk in PBS with 0.1% Tween-20, we incubated the membranes with 1:1,000 anti-HAND1 polyclonal antibody or 1:1,000 anti-HAND2 monoclonal antibody (Abcam, Cambridge, MA, USA); anti-β-actin antibody (Sigma-Aldrich, St Louis, MO, USA; dilution 1:100,000) was used as protein loading control. The proteins were detected by using Enhanced Chemiluminescent detection reagent (Amersham, Piscataway, NJ, USA), according to the manufacturer’s instructions.

### Stimulation with bone morphogenetic protein-4 (BMP4)

Cells were incubated with 5 ng/ml recombinant BMP4 (Peprotech, Rocky Hill, NJ, USA), followed by 100 n*M* pre-miR-363 precursors. Cardiac mesoderm induction was measured by comparing *HAND1* and *HAND2* expression with cells transfected with scrambled miR precursors. Additionally, other growth factors, such as TGF-β, Activin A, and DKK1, were tested for their ability to induce *HAND1* and *HAND2* expression.

### Statistical analysis

All experiments were performed in biological replicates, as indicated, and significance was tested by using the Student *t* test. A *P* value of <0.05 was considered significant.

## Results

### Identification of miRNAs differentially expressed during hESC differentiation and cardiomyocyte-subtype specification

**Figure 1:**
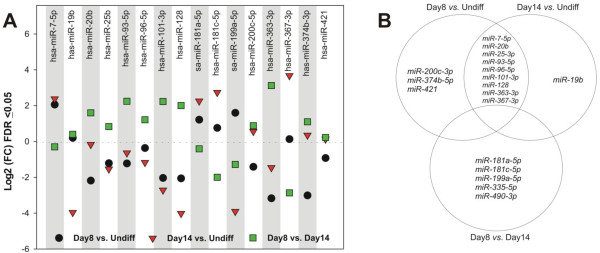
**hESC differentiation into cardiomyocytes identifies developmentally regulated miRNAs. (A)** Cardiac progenitors (Day 8) and embryonic CMs (Day 14) were compared for miRNA expression and represented as log2 fold-change (false discovery rate, FDR, <0.05) of relative miRNA expression compared with undifferentiated cells. **(B)** Venn diagram illustrating miRNAs that were differentially expressed over a 14-day hESC differentiation protocol. Note that the centroid representing undifferentiated versus Day 8 and/or Day 14 analyses contains the miRNAs speculated to target left and right ventricular-specifying transcription factors.

To determine miRNAs that are differentially expressed during differentiation of hESCs into CMs, we performed miRNA expression profiling by using the previously described myocardial reporter hESC line [15]. We compared miRNA expression profiles of undifferentiated hESCs with CMs sorted at Days 8 and 14 after embryoid body (EB) formation [15]. As previously discussed [14], these time points represented the stages at which myocardial precursors and definitive CMs emerge, respectively, from a culture of differentiating hESCs. We previously reported the role of miR-125b in initiating mesoderm formation [14]. In the current study, we analyzed the unique miRNA expression patterns observed in this analysis during early cardiac specification (undifferentiated versus Day 8) and CM subspecialization (Day 8 versus Day 14), as well as the entire arc of CM differentiation (undiff. versus Day 14) (Table 1, see also Additional file 1: Figure S1A). We focused on those novel miRNAs that could potentially target the 3′UTRs of the CM subtype-specific transcription factors, *HAND1*, *HAND2*, *TBX3*, *GJA1*, *NPPA*, *HCN4*, *RYR2*, and *SLN* (Additional file 1: Figure S1B). From >900 known human miRNAs screened, 18 were specifically downregulated more than twofold in hESC-derived CM populations (Figure 1A).

**Table 1:** Differentially expressed miRNAs and predicted targets

	**miRNA**	**Predicted mRNA targets (from TargetScan)**	
		**Total number of mRNA targets**	**mRNA targets specific to cardiac lineage (miRNAs targeting HAND1, HAND2, or NKX2.5)**
*Day 8* v*ersu*s *Undiff.*	137	2031	12 (miR-363, miR-367)
*Day 14* v*ersu*s *Undiff.*	100	1462	10 (miR-363, miR-367)
*Day 8* v*ersu*s *Day 14*	47	142	5 (miR-181a, miR-181c)

To confirm the microarray results, the expression levels of these miRNAs were examined with qPCR. All miRNA expression levels established by qPCR were consistent with microarray data (Additional file 1: Figure S1C). The expression levels of the 18 miRNAs were also assayed in beating tissue microdissected from differentiating hESC cultures after 14 days of differentiation. Interestingly, a subset of miRNAs (miR-363, -367, -181a, -181c) expressed significantly higher levels in hESC-derived non-CMs compared with CMs (Additional file 1: Figure S1C). This subset was predicted to target cardiac transcription factors that regulate CM-subtype specification (Table 2). These results demonstrated that miRNA expression levels vary significantly during hESC differentiation, and that the expression patterns of some miRNAs are restricted to specific tissue types.

**Table 2:** miRNA seed-pairing in cardiac-specific mRNAs

**Symbol (Chr.)**	**Process**	**miR-363**	**miR-367**	**miR-181a**	**miR-181c**	**miR-1**
		**(PcT score)**	**(PcT score)**	**(PcT score)**	**(PcT score)**	**(PcT score)**
GATA6 (18)	Regulate terminal differentiation/proliferation	Np	Np	ACUUACAa (0.52)	ACUUACAa (0.52)	Np
NKX2.5 (5)	Commitment to myocardial lineage	Np	Np	ACUUACAa (0.52)	ACUUACAa (0.52)	Np
HAND1 (5)	Left ventricular cardiac morphogenesis, giant cell differentiation	CACGUUAa (0.78)	CACGUUAa (0.78)	Np	Np	Np
HAND2 (4)	Cardiac morphogenesis, particularly right ventricle and aortic arch	CACGUUAa (0.76)	CACGUUAa (0.76)	ACUUACAa (0.52)	ACUUACAa (0.52)	Np

Np, No pairing.

### *In silico* prediction and validation of miRNAs with predicted binding sites in the 3′UTRs of *HAND1* and *HAND2*

**Figure 2:**
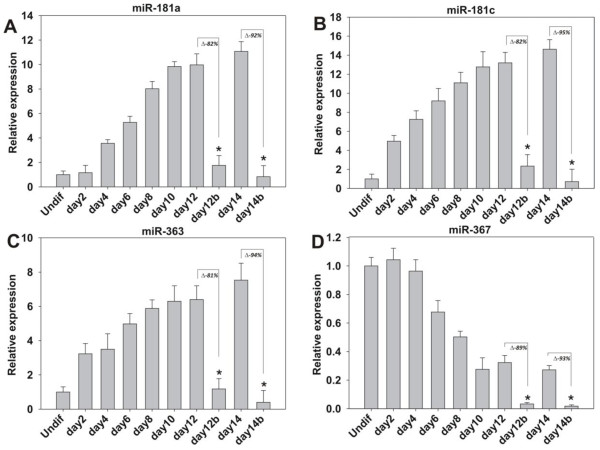
**Endogenous miRNA expression in hESC-derived CMs. (A-D)** Relative expression of miRNAs was assessed with qPCR in undifferentiated (un) and embryoid body-derived CMs. Beating areas representing CMs were isolated on Days 12 and 14. Percentage values reflect differences in expression within cell types. Data shown are mean ± SEM (*n* = 3); *P < 0.05.

TargetScan (version 6.2) was used to search the 3′UTRs of *HAND1* and *HAND2* for the presence of conserved 7/8-mer sites that match the seed regions of each known miRNA. This analysis identified 10 and 20 evolutionarily conserved, predicted miRNA binding sites within the 3′UTRs of *HAND1* and *HAND2*, respectively. A set of six miRNAs (miR-367, -32, -363, -25, -92b, and -92a) were identified that were predicted to bind sites in both genes, with two conserved sites in *HAND1* and one in *HAND2* (see Additional file 2: Figure S2A). MiR-181a and -181c sites were found in *HAND2* only and *NKX2.5* (Table 2). This association between putative miRNA binding sites and *HAND* gene expression was evaluated by using qPCR. Differential expression of *HAND1* and *HAND2* was observed over the course of hESCs differentiation (see Additional file 3: Figure S3A). Likewise, all eight miRNAs showed a steady increase in expression during differentiation; however, beating CMs showed a significant downregulation of all miR-363, -367, -181a, and -181c (Figure 2A-D). The expression of miRNAs was similar in hiPSCs (Additional file 3: Figure S3B-E). hiPSC-derived beating CMs displayed high levels of *HAND* gene expression and low levels of miR-363, -367, -181a, and -181c expression.

### Overexpression of miR-363 specifically downregulates *HAND1* expression in differentiating hESCs

**Figure 3:**
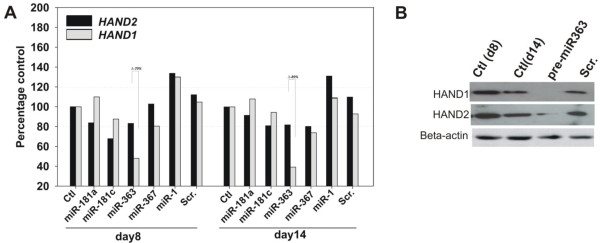
**miR-363-dependent downregulation of *HAND1 *expression.** Pre-miRNAs were transfected into differentiating hESCs by using lipofectamine. **(A)** Cells were assayed for *HAND1* and *HAND2* mRNA expression and **(B)** protein levels. Pre-miR-363 treatment caused a decrease in relative levels of HAND1 mRNA and protein. Percentage values reflect differences in HAND2 and HAND1 expression. Data shown as mean ± SEM (*n* = 3); *P < 0.05.

**Figure 4:**
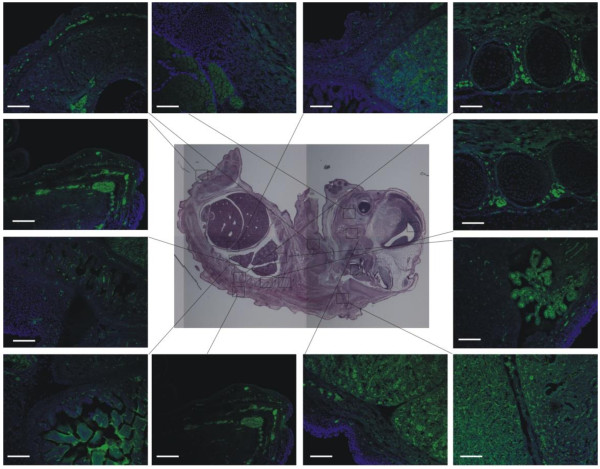
**Analysis of miR-363 expression by F-*ISH *in *E*10.5 mouse embryos.** F-*ISH* was performed with miR-363 antisense and negative control. Fluorescent-green staining indicated positive detection of miR-363 expression in various mouse organs. Attention is called to the exclusion of miR-363 expression from the developing heart. Bar = 100 μm.

To test the association between miRNA expression and target-gene expression, miRNA precursors were transfected into differentiating hESCs, and expression of putative mRNA targets, *HAND1* and *HAND2*, was analyzed with qPCR. Overexpression of pre-miR-363 was associated with significant downregulation of *HAND1* relative to *HAND2* expression (Figure 3A). Pre-miR-367 overexpression was associated with more-modest downregulation of *HAND1* and *HAND2* expression in almost equal proportions (Figure 3A). In contrast, introduction of pre-miR-181a and pre-miR-181c had no detectable effect on *HAND* gene expression (Figure 3A). Because posttranscriptional regulation by miRNAs ultimately affects protein translation, we examined HAND protein expression with immunoblot. Pre-miR-363 overexpression completely abrogated HAND1 protein expression with little effect on HAND2 protein (Figure 3B). We also examined the expression of miR-363 in E10.5 mouse embryos by using fluorescence *in situ* hybridization. Endogenous miR-363 was expressed primarily in brain, limb, liver, pancreas, notochord, and skin, but not heart (Figure 4), consistent with its negative regulatory role in CM differentiation (Figure 3).

### miR-363 inhibits *HAND1* expression through 3′UTR binding

**Figure 5:**
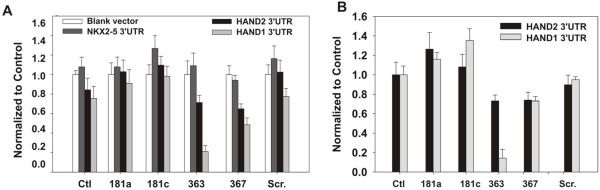
**miR-363 targeting of the 3′UTRs of *HAND1 *and *HAND2*.** Reduced luciferase activity in HEK293 **(A)** and hESCs **(B)** overexpresses pre-miR-363 and *HAND1* or *HAND2* reporters. Plasmids expressing firefly luciferase without 3′UTRs were used as controls. Data shown are mean ± SEM (*n* = 3); *P < 0.05.

We performed dual-luciferase reporter assays to investigate the functional interaction between miR-363 and the 3′UTR of *HAND1* and *HAND2*. The full-length 3′UTR containing the predicted miR-363/miR-367 recognition elements present in *HAND1* (two sites) and *HAND2* (single site) were inserted downstream of the firefly luciferase cDNA in pEZX-MT01 (Additional file 2: Figure S2B). These vectors also express *Renilla* luciferase as a control for transfection. Overexpression of pre-miR-363 repressed luciferase activity in both hESCs and HEK293 cells co-transfected with *HAND1* and *HAND2* 3′UTR reporters. Luciferase inhibition was more robust in cells transfected with the *HAND1* reporter compared with cells expressing the *HAND2* reporter (Figure 5). In contrast, luciferase activity from both reporters was equally affected by pre-miR-367 overexpression (Figure 5). In contrast, luciferase activity from either reporter was not affected by pre-miR-181a or pre-miR-181c expression. We also performed reporter assays by using an NKX2.5 3′UTR reporter, and found that none of the four pre-miRNAs affected luciferase activity (Figure 5). These data confirmed the presence of functional miR-363 binding sites(s) in the 3′UTRs of both human *HAND1* and *HAND2*, suggesting that the changes in *HAND* gene expression with miR-363 overexpression resulted from binding of miR-363 to the 3′UTRs of these genes.

### miR-363 regulates BMP4-mediated *HAND* gene expression during cardiomyocyte differentiation

**Figure 6:**
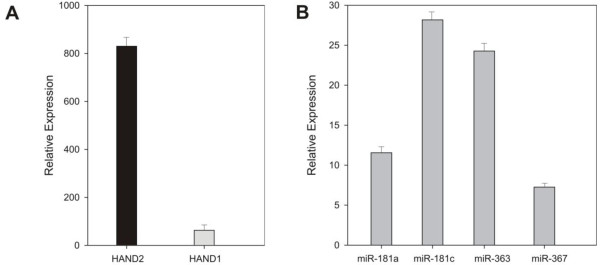
**Relative expression of *HAND2 *mRNA-miR-363 on BMP4 stimulation. (A)** Short exposure to BMP4 showed significant increase in *HAND2* expression levels. **(B)** miR-363 expression was significantly downregulated with BMP4 treatment, as assessed by qPCR. Data shown are mean ± SEM (*n* = 3); *P < 0.05.

We tested various growth factors known for their roles in routing mesoderm formation. Incubation with 5 ng/ml of BMP4 significantly induced *HAND2* mRNA (3.2-fold) compared with *HAND1* and nontreated cell controls (Figure 6A). In contrast, cells exposed to TGF-β, Activin A, DKK1, or WNT3a showed no effect in modulating the expression of *HAND1/2* genes (Additional file 4: Figure S4A). We also analyzed the endogenous expression of these four miRNAs. BMP4 stimulation also induced the expression of endogenous miR-363 and miR-181c, but not miR-367 or miR-181a (Figure 6B).

**Figure 7:**
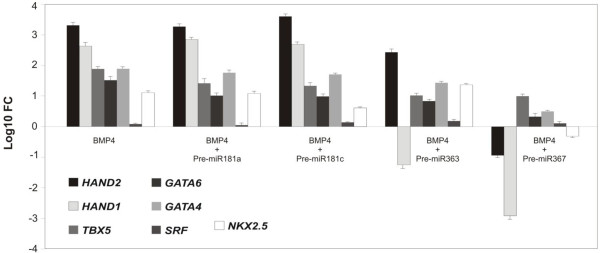
**miR-363 selectively inhibits *HAND1 *expression.** hESCs pretreated with BMP4 were transfected with pre-miR-363. Relative expressions of cardiac-specific transcription factors were analyzed by qPCR. *HAND1* mRNA levels decreased with addition of pre-miR-363, relative to *HAND2* and other transcription factors. Data shown are mean ± SEM (*n* = 3); *P < 0.05.

We next examined the expression of cardiac-specific transcription factors with BMP4 induction in the presence of overexpressed, exogenous, pre-miRNA precursors. Pre-miR-363 overexpression significantly reduced *HAND1* expression compared with *HAND2*. In contrast, pre-miR-367 overexpression resulted in the inhibition of both *HAND* genes, suggesting that miR-367 is not specific to *HAND1*. This conclusion is supported by the differential response of *HAND1* and *HAND2* to BMP4, which has been shown selectively to downregulate *HAND1* in conjunction with overexpression of pre-miR-363. This indicates that the effect can be attributed primarily to miR-363-*HAND1* mRNA interactions. Overexpression of pre-miR-363 had no effect on expression of any of the other cardiac transcription factors tested, and overexpression of pre-miR-181a or -miR-181c had no effect on any of the cardiac transcription factors tested (Figure 7).

### Anti-miR-363 directs the differentiation of *HAND1*-enriched cardiomyocytes

**Figure 8:**
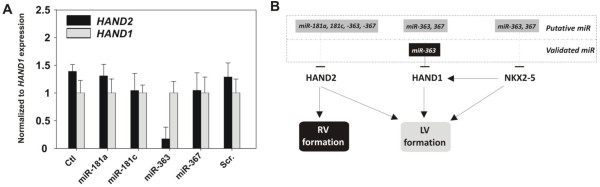
**Cardiac cell fate with anti-miR-363 treatment.** hESCs were differentiated in the presence of anti-miR-363, and beating clusters were microdissected and analyzed with qPCR. **(A)***HAND1*-enriched CMs were produced with miR-363 inhibition. Data shown are mean ± SEM (*n* = 3); *P < 0.05. **(B)** Schematic representation of miR-363 function. miR-363 regulates *HAND1* expression, independent of NKX2.5, leading to left ventricular CM-subtype specification.

In light of the preceding data, we used miRNA inhibition to define further their role in hESC differentiation toward the cardiac lineage. On Day 14 of hESC differentiation, individual beating areas were microdissected and assayed for *HAND1/2* expression by using qPCR. Overexpression of anti-miR-363 markedly increased the number of *HAND1*-expressing CMs (Figure 8A). These data demonstrate that inhibition of miR-363 results in upregulation of *HAND1* translation and enriches a left ventricular cell population.

## Discussion

MiRNAs are known to regulate gene expression in various organs and are recognized as important regulators of cardiac development and function [16,17]. The cardiac-specific transcription factors, *HAND1* and *HAND2,* play important roles in left versus right ventricular determination. We tested a subset of differentially expressed miRNAs predicated to target CM subtype-specifying transcription factors, such as *HAND1* and *HAND2. In vivo* regional expression, *in silico* predictions, and experimental validation demonstrated that miR-363 is an upstream regulator of *HAND1* translation, leading to a role in left ventricular CM differentiation.

To our knowledge, regulation of *HAND1* or *HAND2* by miRNAs has not previously been reported. These two genes are closely related and display complementary and overlapping expression patterns in the developing heart [18,19]. During development, *HAND1* is expressed in the inflow segment of the linear heart tube destined to become the left ventricle, whereas *HAND2* is expressed much earlier throughout the linear heart tube, and then later expressed in the outflow portion of the heart tube destined to become the right ventricle and atria [20]. However, little is known about the posttranscriptional control of *HAND* gene expression, although conserved miRNA seed-pairing sequences in the 3′UTRs of both *HAND1* and *HAND2* suggest control by miRNAs. In this study, we sought to identify specific miRNAs that target specifically ventricular CM-determining genes. The expression profiles of known human miRNAs were analyzed to identify potential miRNA-mRNA interactions that effect hESC differentiation into CMs.

miRNAs are known to target mRNAs by imperfect base pairing with their 3′UTR [21]. This in turn inhibits translation and/or destabilizes the targeted mRNA [22], ultimately controlling its expression. Here, we identified subsets of 137, 100, and 47 miRNAs that were highly expressed in day 8 CM precursors versus undifferentiated cells, day 14 CMs versus undifferentiated cells, and Day 8 CM precursors versus Day 14 CMs, respectively (Table 1). The left/right ventricle transcriptional determinants, *HAND1* and *HAND2,* were identified as targets of four miRNAs, (miR-363, -367, -181a, and -181c).

In a previous report [14], we showed that miR-125b regulated the development of hESC-derived early mesoderm and was highly expressed in cardiac precursors. We showed that miR-125b targets *Lin28*, indirectly inhibits *Nanog* and *Oct4,* and promotes onset of *Brachyury*, *GATA4,* and *NKX2.5* expression to induce cardiac mesoderm formation. In both this and the current study, we predicted the target genes based on conserved pairing regions [21]. We are aware that the majority of these predictions rely on extensive complementarity, while accounting for other features that contribute to miRNA 3′UTR recognition [23-26].

hESC differentiation *in vitro* leads to multiple cell lineages arising from the three embryonic germ layers, including CMs. The spontaneous differentiation of hESCs into CMs, however, leads to a heterogeneous mixture of CMs [3,10]. Although several protocols are used for inducing CM differentiation, we used a method that allows ongoing interaction between many CM subtypes [10]. In addition, we used a previously described aMHC-EGFP reporter hESC line that allowed us to sort specifically cardiac precursors and embryonic CMs [15].

The expression of miR-363 was detected with fluorescence *in situ* hybridization in *E*10.5 mouse embryos. miR-363 was expressed in the developing limb bud, notochord, ectoderm, and brain. These results are consistent with the miR-363 expression in chick embryo reported previously [27], in which miR-363 was observed in ectoderm, pharyngeal arches, notochord, and limb bud, suggestive of wide function in limb development, patterning, and central nervous system development. Tissue-specific miRNA expression implies a negative regulatory role in expression f their targets. However, many transcription factors demonstrate more-complicated, stoichiometric expression during the course of tissue development. Relevant to this study, *HAND2* is initially expressed throughout the developing heart tube, but then is restricted to second heart-field structures, with *HAND1* expression restricted to the developing left ventricle [20]. The mechanisms responsible for this developmentally regulated expression of *HAND* genes has until now been elusive.

Two evolutionarily conserved miR-363 seed-pairing sites in human *HAND1* 3′UTR suggested that the miRNA pairing sequences predicted *in silico* contribute to *HAND1* regulation. *HAND1* 3′UTR reporter activity was completely abolished by miR-363 in contrast to *HAND2* 3′UTR activity. NKX2.5 also has been implicated in left/right asymmetric expression of *HAND1* and *HAND2*[28]; however, a role for miR-363 in regulating NKX2.5 expression was not suggested by our results.

BMP signaling controls the differentiation of CMs in multiple ways [29]. In this work, BMP4 or BMP2 was shown to elicit induction of CM differentiation not only from precardiac mesoderm but also from tissue that is normally not cardiogenic. We tested various signaling molecules that have been implicated in cardiac induction [30-32]. TGF-β family members such as Activin A, bFGF, and BMPs, have been identified as promoting the terminal differentiation of precardiac mesoderm; however, when used at concentrations reported to be pharmacologically effective, neither Activin A nor bFGF displayed any discernible *HAND1/2*-inducing effect. BMP4 is closely related to the TGF-β family member BMP2, is expressed in anterior lateral ectoderm, and is indistinguishable from BMP2 in cardiac-inducing activity [29]. We observed similar induction of *HAND1* and *HAND2* with BMP4 and BMP2. Taken together, these findings suggested a model for BMP-mediated cardiac induction and CM-subtype specification through miR-363 and differential expression of *HAND1/2* (Figure 8B).

CM commitment after differentiation of hESCs was studied in the presence of a miR-363 antagomir. The isolated cardiogenic cells overexpressing anti-miR-363 expressed the left-specifying factor *HAND1* at levels significantly higher than control. Cardiac progenitors that differentiate *in vitro* accumulate muscle-specific proteins but do not necessarily exhibit conspicuous beating or cross-striations; thus, these may be overlooked with less-sensitive assays with histology or spontaneous contractility.

We used qPCR to assess the differences in miRNA expression between beating foci and evaluated the ratio of *HAND1* to *HAND2* genes. miRNAs are known to act as transcriptional repressors of their target RNAs, thereby downregulating gene expression. However, it is possible that critical regulatory proteins may compensate for posttranscriptional downregulation by other mechanisms (for example, increased protein stability, decreased protein turnover). Therefore, our analysis may not identify every regulatory feature of the miRNA-CM subtype-specification pathway.

Right-ventricle CMs were devoid of *HAND1* expression, which allowed us to distinguish between left and the right ventricular CMs. One explanation could be that the emergence of CMs from committed progenitors is influenced by cell density. High densities of committed progenitor cells show distinct populations of atrial and ventricular CMs [33]. It appears that differentiation in the presence of anti-miR-363 causes progenitor cells to differentiate into a *HAND1*-rich population, and left-ventricular CMs constitute the greatest percentage of cells expressing *HAND1*.

## Conclusion

Our results demonstrate for the first time that miR-363 plays an important role in posttranscriptional regulation of CM differentiation by targeting *HAND1*. These findings elucidate the mechanism by which differential *HAND* gene expression is achieved during cardiac development at the cellular level, and may be a valuable strategy for generating left ventricular CMs for further basic study and cell-therapy applications.

## Abbreviations

BMP4: Bone morphogenetic protein 4; CDNA: complementary DNA; CMs: cardiomyocytes; DKK1: Dickkopf-1; EB: embryoid body; FDR: false discovery rate; FGF-2: fibroblast growth factor 2; FISH: fluorescence *in situ* hybridization; GAPDH: glyceraldyde 3-phosphate dehydrogenase; HAND1: hand and neural crest derivative expressed 1; hESC: human embryonic stem cell; hIPSC: human induced pluripotent stem cell; LNA: locked nucleic acid; miRNA/miR: microRNA; PBS: phosphate-buffered saline; qPCR: real-time quantitative polymerase chain reaction; TBST: Tris-buffered saline and 10% Tween 20; TGF-β: transforming growth factor-β; UTR: untranslated region.

## Competing interests

The authors declare that they have no competing interests relevant to this work. HSB is a current employee of Merck Sharpe & Dohme Corp., a subsidiary of Merck & Co., Inc., and may own stock or hold stock options in the company.

## Authors’ contributions

VW designed and performed experiments, analyzed data, and wrote the manuscript; AP, KW, and SP performed experiments and analyzed data; HSB designed experiments, analyzed data, and wrote the manuscript. All authors discussed the results and their implications, and they approved the final manuscript.

## Supplementary Material

Additional file 1: Figure S1. Selection of miRNAs that control CM-subtype specification. **(A)** miRNA expression patterns were used to categorize miRNAs into pathways that specify cardiac mesoderm or subsequent CM subtype. **(B)** Major CM subtypes and associated genes are depicted. **(C)** Validation of miRNA microarray data by qPCR. Click here for file

Additional file 2: Figure S2. Reporter vectors used to interrogate *HAND1* and *HAND2* 3′UTR binding. **(A)** Evolutionarily conserved predicted miRNA binding sites in the 3′UTRs of human *HAND1* and *HAND2*. **(B)** Schematic representation of the luciferase reporter vectors containing the full-length 3′UTRs. Click here for file

Additional file 3: Figure S3. Comparison of miRNA expression patterns in differentiating hESCs and hiPSCs. **(A)** Relative expression of mRNA in hESC-derived cells. **(B)** Relative miRNA expression in hiPSC-derived cells. Data shown are mean ± SEM. (*N* = 3); *P < 0.05. Click here for file

Additional file 4: Figure S4. Effects of growth-factor stimulation on mRNA and miRNA expression. Relative expression of mRNA **(A)** and miRNA **(B)** with stimulation by indicated growth factors. Data shown are mean ± SEM. (*N* = 3); *P < 0.05. Click here for file
