# Supplementary figures and images for: MicroRNA-363 negatively regulates the left ventricular determining transcription factor HAND1 in human embryonic stem cell-derived cardiomyocytes
Source: Stem Cell Res Ther. 2014 Jun 6;5(3):75. doi: 10.1186/scrt464 (PMC4097848; doi:10.1186/scrt464)

## Slide 1
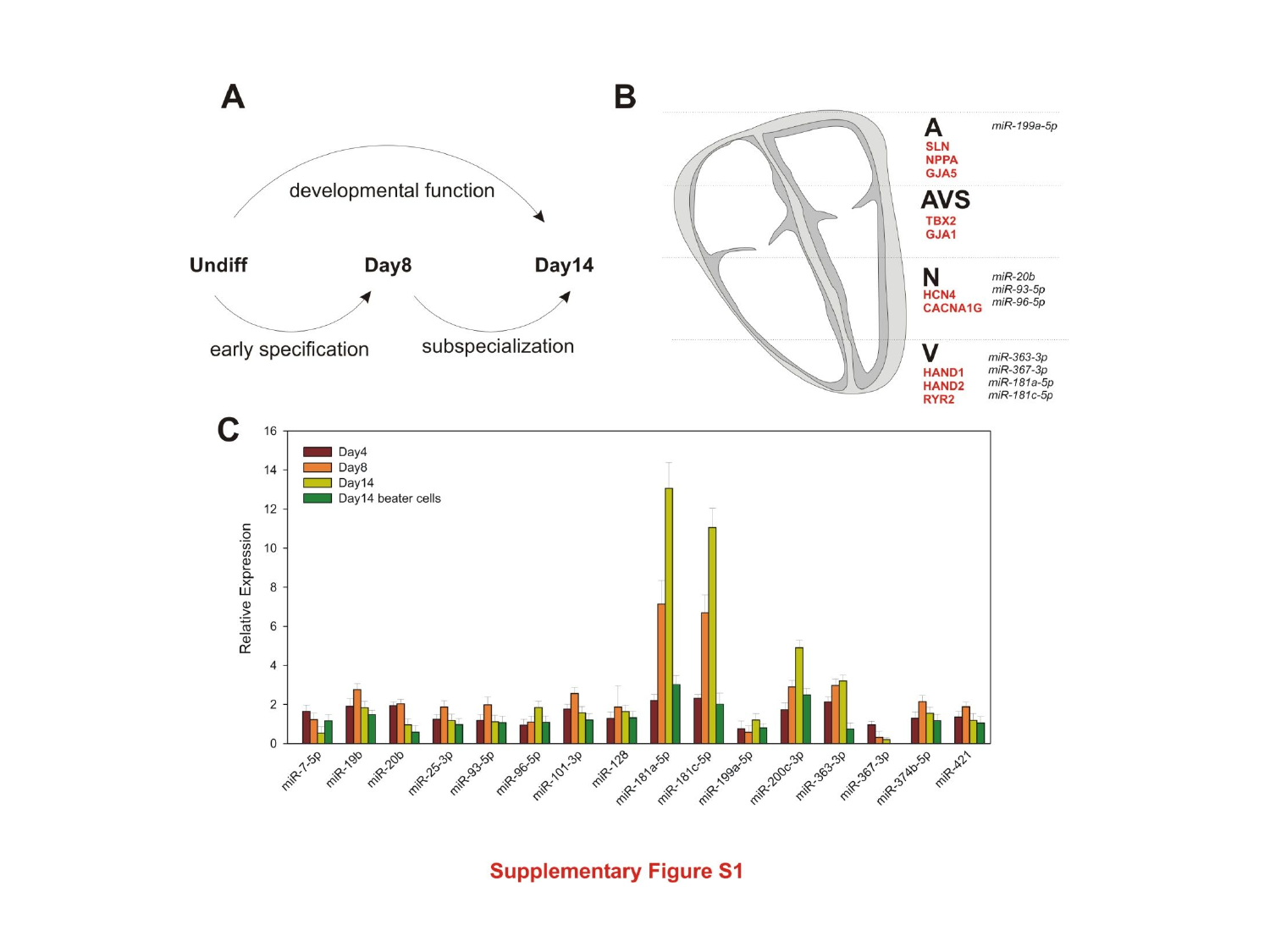

Supplement: Additional file 1: Figure S1. — Selection of miRNAs that control CM-subtype specification. (A) miRNA expression patterns were used to categorize miRNAs into pathways that specify cardiac mesoderm or subsequent CM subtype. (B) Major CM subtypes and associated genes are depicted. (C) Validation of miRNA microarray data by qPCR. [file scrt464-S1.ppt]

## Slide 1
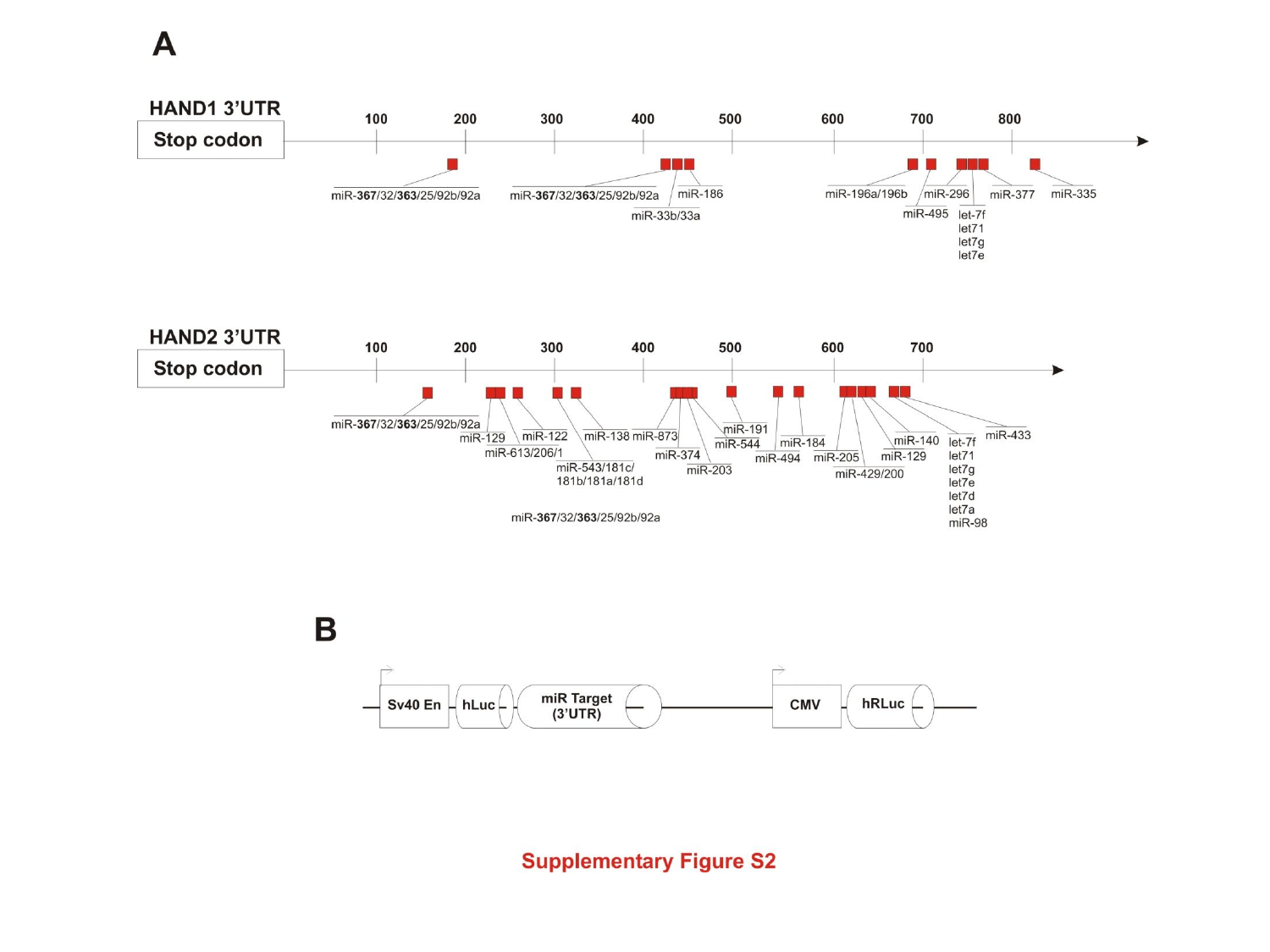

Supplement: Additional file 2: Figure S2. — Reporter vectors used to interrogate HAND1 and HAND2 3′UTR binding. (A) Evolutionarily conserved predicted miRNA binding sites in the 3′UTRs of human HAND1 and HAND2. (B) Schematic representation of the luciferase reporter vectors containing the full-length 3′UTRs. [file scrt464-S2.ppt]

## Slide 1
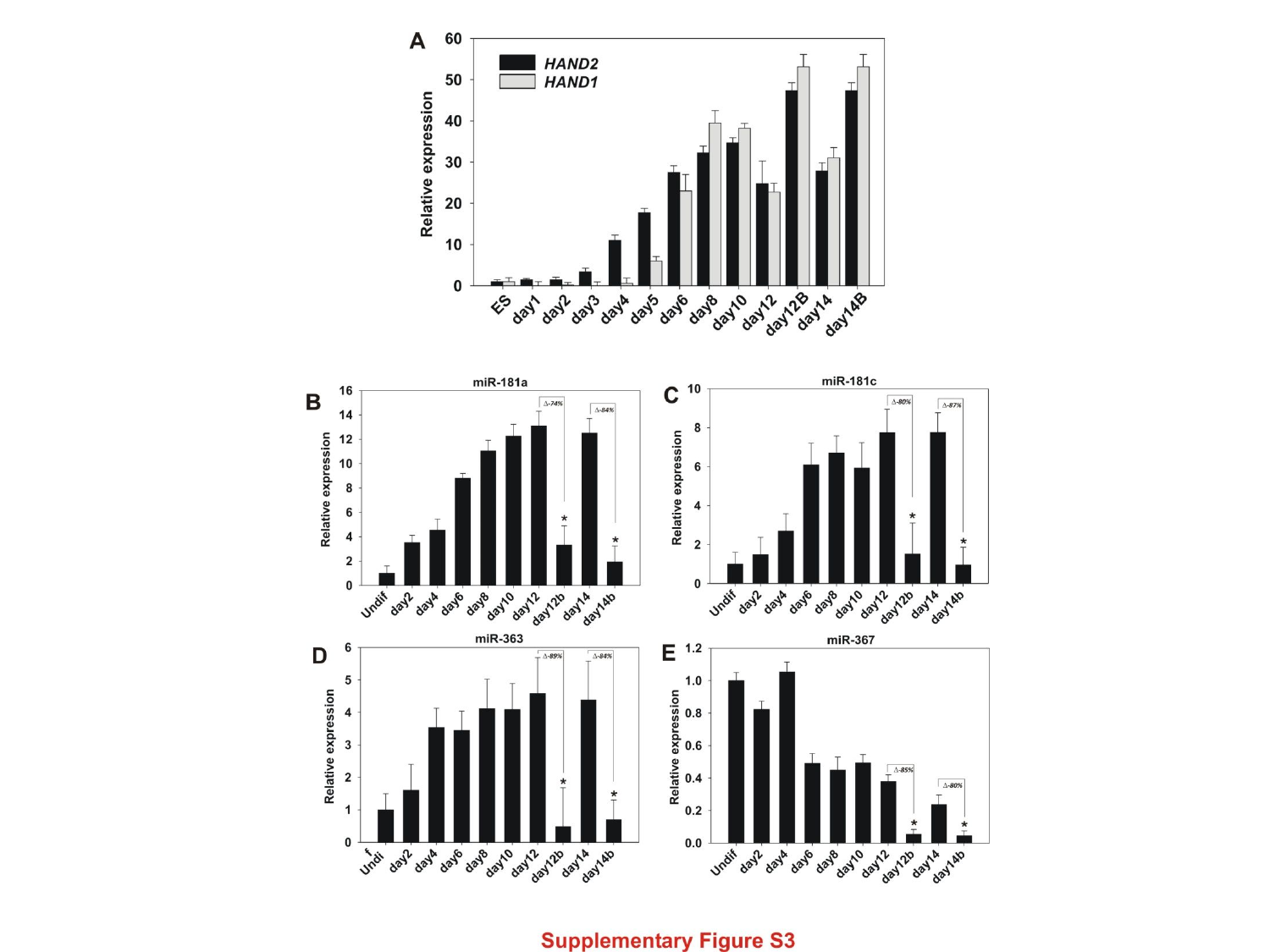

Supplement: Additional file 3: Figure S3. — Comparison of miRNA expression patterns in differentiating hESCs and hiPSCs. (A) Relative expression of mRNA in hESC-derived cells. (B) Relative miRNA expression in hiPSC-derived cells. Data shown are mean ± SEM. (N = 3); *P < 0.05. [file scrt464-S3.ppt]

## Slide 1
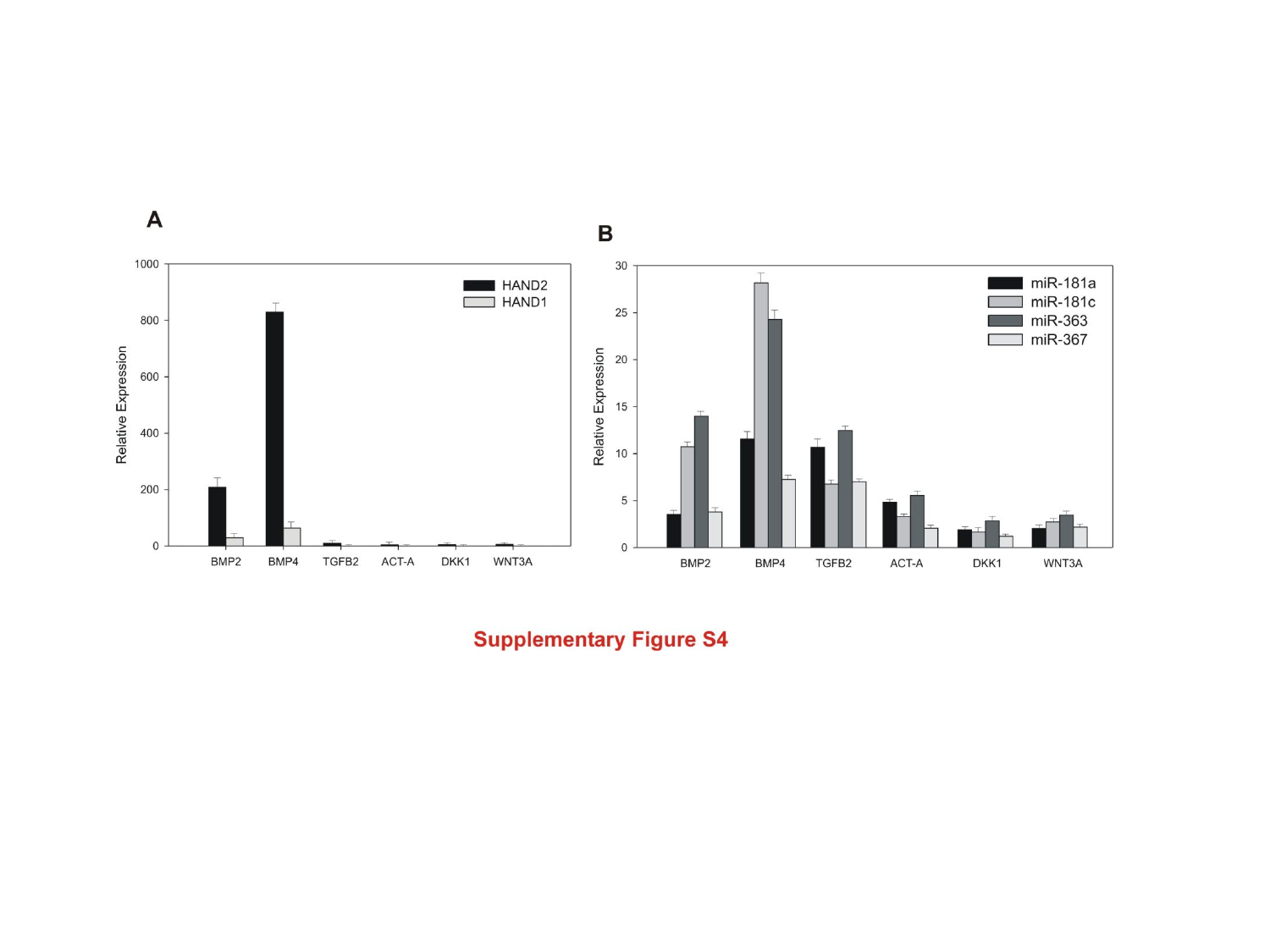

Supplement: Additional file 4: Figure S4. — Effects of growth-factor stimulation on mRNA and miRNA expression. Relative expression of mRNA (A) and miRNA (B) with stimulation by indicated growth factors. Data shown are mean ± SEM. (N = 3); *P < 0.05. [file scrt464-S4.ppt]
